# Futsal Match-Related Fatigue Affects Running Performance and Neuromuscular Parameters but Not Finishing Kick Speed or Accuracy

**DOI:** 10.3389/fphys.2016.00518

**Published:** 2016-11-07

**Authors:** Fabio Milioni, Luiz H. P. Vieira, Ricardo A. Barbieri, Alessandro M. Zagatto, Nikolai B. Nordsborg, Fabio A. Barbieri, Júlio W. dos-Santos, Paulo R. P. Santiago, Marcelo Papoti

**Affiliations:** ^1^Post Graduate Program in Movement Science, Department of Physical Education, UNESP - Univ Estadual PaulistaRio Claro, Brazil; ^2^School of Physical Education and Sports of Ribeirão Preto, University of São PauloRibeirão Preto, Brazil; ^3^Post Graduate Program in Rehabilitation and Functional Performance, Faculty of Medicine at Ribeirão Preto, University of São PauloRibeirão Preto, Brazil; ^4^Department of Physical Education, UNESP - Univ Estadual PaulistaBauru, Brazil; ^5^Department of Nutrition, Exercise and Sport, University of CopenhagenCopenhagen, Denmark

**Keywords:** automatic tracking, twitch Interpolation, fatigue, EMG, exercise physiology, sport performance

## Abstract

**Purpose:** The aim of the present study was to investigate the influence of futsal match-related fatigue on running performance, neuromuscular variables, and finishing kick speed and accuracy.

**Methods:** Ten professional futsal players participated in the study (age: 22.2 ± 2.5 years) and initially performed an incremental protocol to determine maximum oxygen uptake ($\dot{\text{V}}{\text{O}}_{2\text{max}}$: 50.6 ± 4.9 mL.kg^−1^.min^−1^). Next, simulated games were performed, in four periods of 10 min during which heart rate and blood lactate concentration were monitored. The entire games were video recorded for subsequent automatic tracking. Before and immediately after the simulated game, neuromuscular function was measured by maximal isometric force of knee extension, voluntary activation using twitch interpolation technique, and electromyographic activity. Before, at half time, and immediately after the simulated game, the athletes also performed a set of finishing kicks for ball speed and accuracy measurements.

**Results:** Total distance covered (1st half: 1986.6 ± 74.4 m; 2nd half: 1856.0 ± 129.7 m, *P* = 0.00) and distance covered per minute (1st half: 103.2 ± 4.4 m.min^−1^; 2nd half: 96.4 ± 7.5 m.min^−1^, *P* = 0.00) demonstrated significant declines during the simulated game, as well as maximal isometric force of knee extension (Before: 840.2 ± 66.2 N; After: 751.6 ± 114.3 N, *P* = 0.04) and voluntary activation (Before: 85.9 ± 7.5%; After: 74.1 ± 12.3%, *P* = 0.04), however ball speed and accuracy during the finishing kicks were not significantly affected.

**Conclusion:** Therefore, we conclude that despite the decline in running performance and neuromuscular variables presenting an important manifestation of central fatigue, this condition apparently does not affect the speed and accuracy of finishing kicks.

## Introduction

Futsal is characterized by high-intensity intermittent exercises with short recovery periods (Castagna et al., 2009; Caetano et al., 2015), with 5 and 12% of the total playing time being sprinting and high-intensity running, respectively (Barbero-Alvarez et al., 2008). Castagna et al. (2009) reported that Spanish athletes covered 121 m.min^−1^, performed a sprint every ~79 s, reached 75% of maximal oxygen uptake ($\dot{\text{V}}{\text{O}}_{2\text{max}}$), 90% of maximum heart rate and a mean blood lactate concentration of ~5.3 mmol.L^−1^ (Castagna et al., 2009).

The high physical demands of futsal induce match-related fatigue, resulting in impaired performance, as evidenced by a gradual reduction in total distance covered (Castagna et al., 2009; De Oliveira Bueno et al., 2014) and an increase in distance covered at speeds below 6 km.h^−1^ (i.e., walking and repositioning activities) (De Oliveira Bueno et al., 2014). Additionally, the torque of knee extension is reduced in a time-dependent relationship (Dal Pupo et al., 2014). Based on the high-intensity nature of the game and the gradual reduction in running performance, it appears likely that kick efficiency may be compromised as the game progresses.

While previous studies have investigated the futsal finishing kick (Barbieri et al., 2010, 2015), to our knowledge there are no studies that have investigated the influence of fatigue on kick performance during futsal games and the few studies regarding soccer simulations present contradictory results. Basically, accuracy (Ali et al., 2007) and ball speed (Russell et al., 2011; Radman et al., 2016) have been found to be compromised by fatigue development, however this may be counteracted by some strategies, such as the ingestion of carbohydrate rich drinks (Currell et al., 2009).

Muscle fatigue may not only affect the technical performance, but can also alter repeated-sprint ability (RSA) performance, which has been linked with in-match performance alterations (Rampinini et al., 2008, 2011). In top level Italian soccer players, match-related fatigue was manifested as a combination of central and peripheral factors, wherein central fatigue presented a moderate/strong relationship with isometric force of the muscle quadriceps and sprint performance, whereas peripheral fatigue was associated with muscle soreness (Rampinini et al., 2011). Noteworthy, increasing muscular fatigue appears to be associated with compromised technical performance (short-pass ability) (Rampinini et al., 2008).

These observations indicate that the development of fatigue in futsal may result in neuromuscular fatigue with potential implications for technical performance. However, this hypothesis has never been addressed. Therefore, the aim of this study was to investigate the influence of futsal match-related fatigue on running performance, neuromuscular variables and finishing kick speed and accuracy. We hypothesized that the fatigue process induced by futsal would increase the physiological strain as well as reduce running performance and impair neuromuscular variables (i.e., central and peripheral neuromuscular fatigue) affecting the finishing kick speed and accuracy.

## Methods

### Participants

Ten professional outfield futsal players (age: 22.2 ± 2.5 years; body mass: 72.7 ± 8.5 kg; height: 174 ± 7 cm; $\dot{\text{V}}{\text{O}}_{2\text{max}}$: 50.6 ± 4.9 mL.kg^−1^.min^−1^) belonging to a team that competed in the first division of the São Paulo Futsal State Championship participated voluntarily. The athletes presented no history of muscle disorders, had professional competitive experience of at least 4 years (i.e., state and national championship leagues), and generally participated in ~8 training sessions, lasting ~100 min each and one official game per week. The procedures were in accordance with the Helsinki declaration and were previously approved by the local Research Ethics Committee.

### Experimental design

**Figure 1:**
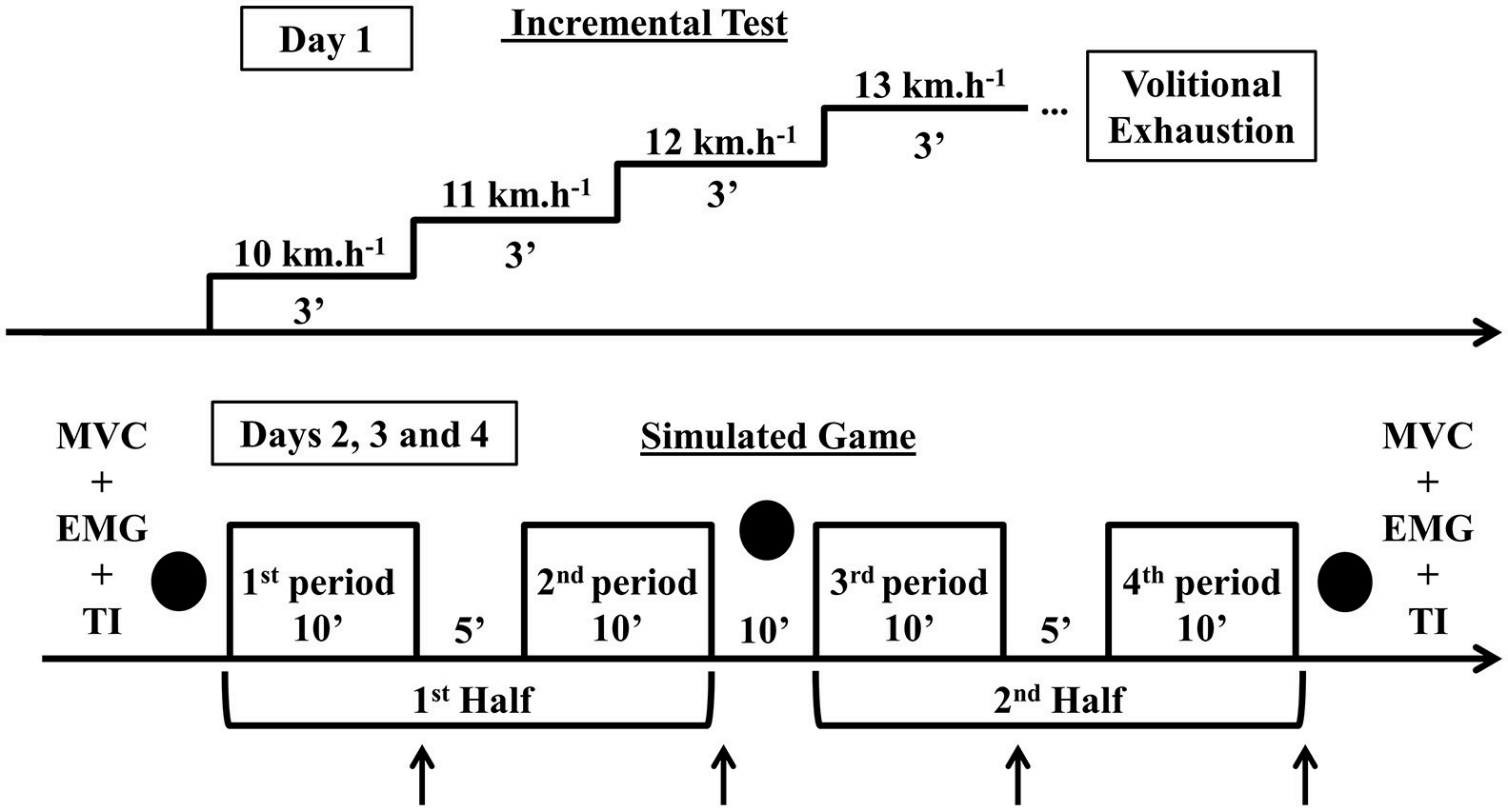
**Diagrammatic representation of the protocol**. ↑, Blood samples; •, Sets of kicks; MVC, Maximal isometric voluntary contraction; EMG, Surface electromyography; TI, Twitch interpolation technique.

Initially participants performed an incremental test on a motorized treadmill until exhaustion. On separate days they participated in three simulated games that were video recorded for later computer-based automatic tracking and running performance analysis. The mean (min–max) environmental air temperature and relative humidity of the period in which each simulated game was carried out were 30.9°C (28.7–32.1°C) and 49.7% (41.6–59.8%) on the first day; 32.0°C (29.7–32.4°C) and 44.4% (40.9–50.8%) on the second day; and 27.0°C (23.4–27.3°C), and 46.7% (43.0–55.2%) on the third day (climate records from the Institute of Meteorological Research–IPMet–Bauru–SP–Brazil). Prior to the simulated games, all participants were familiarized with the procedures, especially the set of finishing kicks and neuromuscular assessments. Finishing kick performance was evaluated through three sets of three 10-m kicks allocated before the beginning (i.e., baseline), at half-time, and immediately after the simulated games. Four players were evaluated on the first day and three players on the second and third days of the simulated games. The number of participants evaluated on each day was limited with the aim of enabling all sets of kicks to be executed in less than 2 min. The neuromuscular evaluations were carried out before the first set of kicks (i.e., to determine the baseline of neuromuscular variables) and immediately after the third set of kicks (i.e., to determine the influence of simulated game in neuromuscular variables) and were composed of two maximal 5-s isometric voluntary contractions (MVC) of knee extension separated by 1 min. Surface electromyography (EMG) was recorded from the *vastus lateralis* muscle and neuromuscular function was assessed by the twitch interpolation technique. During the neuromuscular assessment, two players per day were evaluated. The number of participants evaluated each day was limited to enable termination of all neuromuscular assessments in less than 15 min (set of kicks: ~2 min; neuromuscular assessment of each participant: ~5 min). Blood samples were collected immediately at the end of each period (i.e., blood samples collection lasted ~10 s) to measure the blood lactate concentration. The heart rate was measured continuously (Figure 1).

### Determination of maximal oxygen uptake ($\dot{\text{V}}{\text{O}}_{2\text{max}}$)

The incremental treadmill-running test was initiated at 10 km.h^−1^ with increments of 1 km.h^−1^every 3 min and performed on a motorized treadmill (ATL–Inbramed–Porto Alegre–RS–Brazil) at a 1% inclination, until voluntary exhaustion (Billat et al., 2002). The oxygen uptake ($\dot{\text{V}}{\text{O}}_{2}$) and carbon dioxide ($\dot{\text{V}}{\text{CO}}_{2}$) production were monitored breath-by-breath using an ergospirometer (Metalyzer 3B–Cortex–Leipzig–Germany).

The points obtained were smoothed and interpolated in order to consider 1 value per second (OriginPro 8.0–OriginLab Corporation–Northampton–MA–USA). The $\dot{\text{V}}{\text{O}}_{2\text{max}}$ was assumed as the highest $\dot{\text{V}}{\text{O}}_{2}$ average obtained in the final 30 s of the exercise stage, when at least two of the following criteria were met: (1) $\dot{\text{V}}{\text{O}}_{2}$ stabilization of the final two stages of exercise (range <2.1 mL·kg^−1^·min^−1^); (2) respiratory exchange ratio > 1.1; (3) maximum heart rate > 90% of maximum predicted heart rate (Howley et al., 1995).

### Simulated game, blood lactate and heart rate

The simulated game consisted of two halves of 20 min with a 10 min rest interval between halves. Each half was divided into two periods of 10 min with a 5 min rest interval between periods [adapted from Castagna et al. (2009)]. Prior to starting the simulated games, the athletes performed ~10 min of their habitual warm-up that consisted of short sprints with change of directions and exercises with ball involvement. The players remained on court during the entire simulation but not necessarily in the same team.

Blood samples from the earlobe (25 μL) were collected immediately after the end of each period and analyzed using an electrochemical lactimeter YSI 1500 (Yellow Spring–Ohio–USA). The highest value of blood lactate was assumed as peak of blood lactate concentration ([La]_PEAK_), and the mean value of blood lactate concentration was assumed as the mean blood lactate concentration ([La]_MEAN_). Heart rate was collected continuously beat-by-beat using a heart rate monitor (Polar Team–Polar–Kempele–Finland). The heart rate peak (HR_PEAK_) was assumed as the highest value of heart rate and the mean heart rate (HR_MEAN_) was assumed as the mean value of heart rate.

### Automatic tracking

**Figure 2:**
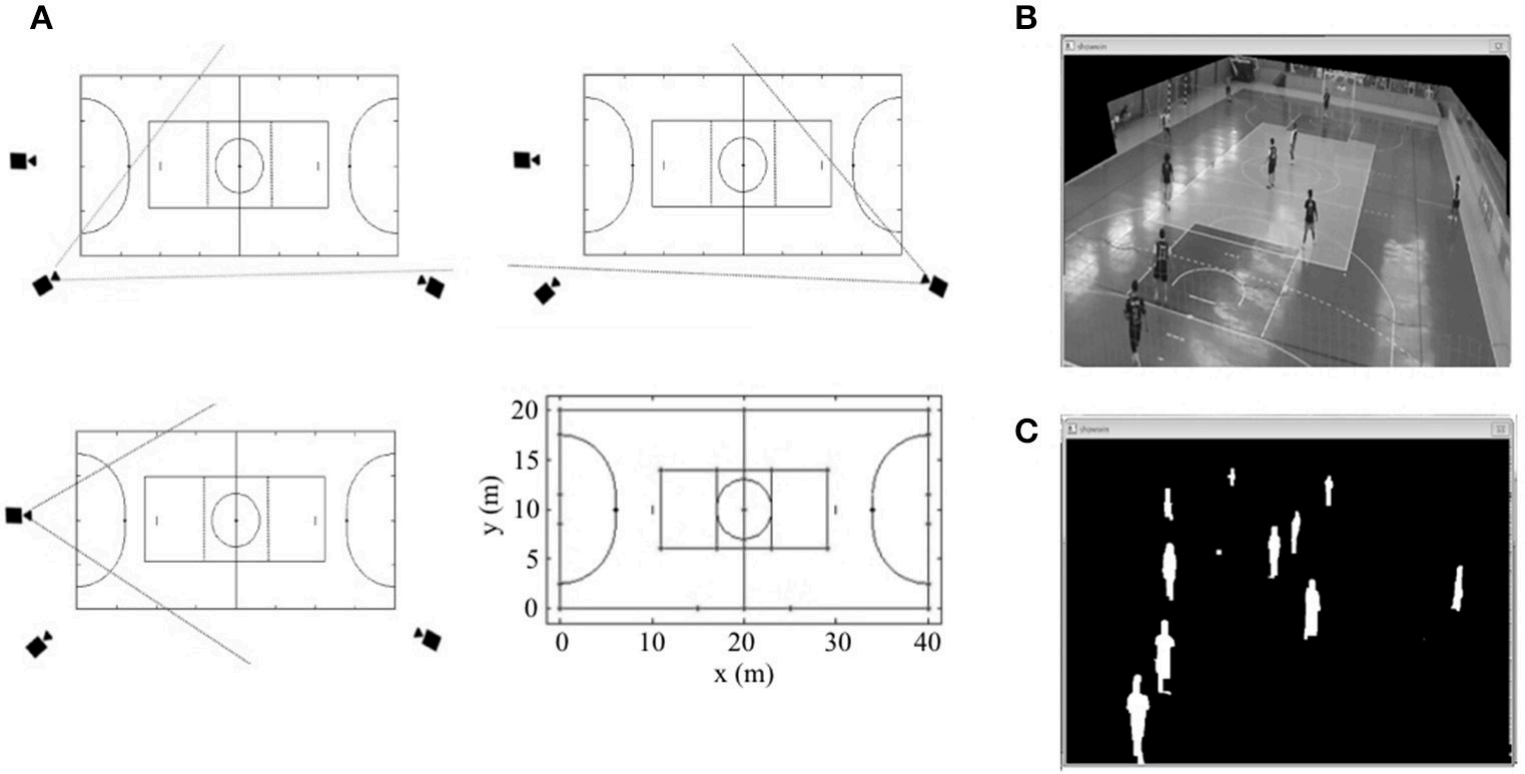
**(A)** Framing area of each video camera and calibration points of the game area used for 2-D calibration. **(B)** Selection of the game area for computational tracking. **(C)** Mathematical segmentation in Dvideow environment.

All simulations were integrally recorded by three digital video cameras (Sony HandCam DCD-SR21–Sony–Tokyo–Japan) adjusted to a frequency of 30 Hz (720 × 480 pixel and 24-bit color resolution), allocated at the highest points of the gym. Subsequently, the images were transferred to a computer and using Dvideow Software (Figueroa et al., 2006; Barros et al., 2007; De Oliveira Bueno et al., 2014), the kinematic procedures were performed (synchronization, calibration, segmentation, tracking and 2-D reconstruction). The coordinate values were later filtered by a 3rd order Butterworth filter, adopting a cut-off frequency of 0.4 Hz (De Oliveira Bueno et al., 2014). In this study the average error for the determination of static positions was 0.14 m and the average error for distance covered was 0.9%. These outcomes were similar to De Oliveira Bueno et al. (2014) and Barros et al. (2007) who used the same software. Using specific algorithms in MatLab (The Math Works Inc–Natick–MA–USA), the running performances were individually calculated: distance covered (DC), distance covered per minute (DC_MIN_), number of sprints (S_N_–every time the athletes exceeded the lowest speed at which the $\dot{\text{V}}{\text{O}}_{2\text{max}}$ was reached) and time spent sprinting (S_T_–∑ of sprints times) (Figure 2).

### Measurements of 10-m stationary finishing kick performance

The kicker order was randomized and followed in all sets. A target of 1 × 1 m was fixed in the center of the goal (Barbieri et al., 2010, 2015) and participants were instructed to perform a maximal ball speed instep kick with the objective of hitting the target (Barbieri et al., 2015).

#### Accuracy

All attempts were recorded by a digital video camera (Casio Exilim EX-F1–Casio–São Paulo–SP-Brazil) adjusted to a frequency of 60 Hz and frontally positioned to the goal. The images of the moment when the ball crossed the goal line were digitized and processed similarly to the images of the players, using Dvideow Software. The accuracy was defined as the Euclidean distance between the position of the ball at the instant that it passed over the goal plan with respect to the nearest point on the target (Barbieri et al., 2015). For accuracy analysis, the mean of the Euclidean distances measured in each attempt in the set was assumed (i.e., the accuracy of set 1 corresponded to the mean of the distances measured in each attempt in set 1).

#### Ball speed measures

To measure the ball speed, all attempts were video recorded by two digital video cameras (Casio Exilim EX-FH25–Casio–São Paulo–SP-Brazil) adjusted to a frequency of 240 Hz, and positioned laterally 2 m from the initial position of the ball. The area was calibrated with rigid material (1 × 1 × 1 m) containing 10 3-D points of known coordinates. The y axis was horizontal (pointing to the goal center), the z axis vertical (pointing up), and the x axis the cross product of y and z (pointing laterally). For speed analysis, the mean of the peak speeds achieved in each attempt in the set was assumed (i.e., the speed of set 1 corresponded to the mean of the peak speeds achieved in each attempt of set 1).

**Figure 3:**
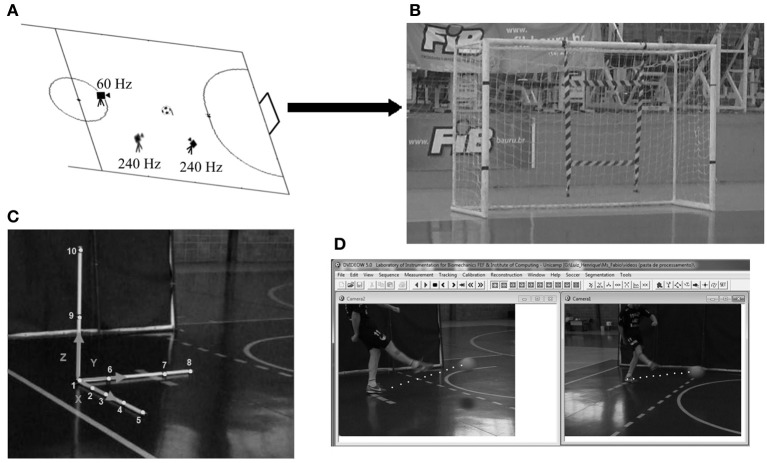
**(A)** Framing area of each video camera for kick speed and accuracy measurements. **(B)** 1 m^2^ goal target. **(C)** Rigid material (1 × 1 × 1 m) used for 3-D calibration of kick area. **(D)** Measurements of 3-D ball speed in Dvideow environment.

Subsequently, the images were transferred to a computer; for conversion to AVI format, synchronization of the two cameras using an audio-band method (Barros et al., 2007), calibration, manual measurement of the ball centroid (Barbieri et al., 2015), and 3-D reconstruction of image sequences, using Dvideow Software (Figueroa et al., 2003). Ten frames were considered after the contact of the ball with the foot. The horizontal components (x- and y-axes) were calculated as the first derivative of linear regression lines, fitted to their non-filtered displacements. The vertical component (z-axis) of the ball was calculated as the first derivative of a quadratic regression line, with its second derivative set equal to −9.81 m.s^−1^ and fitted to its non-filtered displacement in the available airborne frames (Nunome et al., 2006; Barbieri et al., 2010, 2015). An accuracy test was performed (Barbieri et al., 2010) in which a rigid bar containing two markers (20 mm) with a known distance between them (500 mm) was randomly moved through the calibration area during 5500 frames. The calculated accuracy was 6.09 mm. Regarding kicking accuracy calculations, the ten control points used for 3-D calibration of the goal plane were measured three times each on different days. When comparing the reconstructed vs. the expected values, the average error was 0.02 ± 0.01 m (Figure 3).

### Neuromuscular assessment

#### Force data acquisition

Participants performed two 5-s knee extension MVCs with a 1 min interval between them. The participants were positioned in a specially designed chair maintaining knee and hip flexion at 90° (0°Full extension). An inextensible wire was attached to the ankle of the dominant leg and connected to a load cell (CSR-1T–MK Control–São Paulo–SP-Brazil). The load cell signal was acquired by an analog acquisition module (NI 6009–NI Instruments–Austin–TX–USA) with a sample rate of 1000 Hz, and digitally filtered by a fourth-order Butterworth filter with a 15 Hz cutoff frequency, assumed after residue analysis of the signal. The MVC peak force (F_PEAK_) was assumed as the mean of 100 ms during the force plateau.

#### Twitch interpolation

Square-wave doublet electrical pulses (100 Hz–1 ms pulse duration, 10 ms interval between pulses) were delivered at the most sensitive sites of the femoral triangle (cathode) and the gluteal fold (anode) by a high-voltage electrical stimulator (Bioestimulador 200 V peak-to-peak–Insight–Ribeirão Preto–SP–Brazil) through carbon-rubber electrodes (6 × 5 cm). The electrode sites were ink demarked to allow placement reproducibility throughout the experiment. The optimal intensity of stimulation was determined by the application of consecutive incremental doublets to the relaxed muscle until reaching the twitch force plateau (concomitantly with maximal M-Wave amplitude) (Girard et al., 2013) and/or the discomfort limit, however the amperage achieved at the discomfort limit was above the amperage that elicited the twitch force plateau for all participants. The maximal electrical current achieved was assumed and supramaximal stimulation was ensured by increasing the final intensity by 10% (Scaglioni and Martin, 2009).

The amplitude in force signal of the doublet applied to the relaxed muscle prior to MVC (i.e., about 5 s prior) was assumed as the twitch control (TC). Doublets were also delivered at 2–3 s of the MVC and the amplitude over the force plateau was assumed as twitch superimposed (TS). The voluntary activation level was calculated as VA = [1 – (TS/TC)] × 100, however when TS was elicited slightly before or after the MVC force plateau a correction was applied VA = [1 - (TS × (force level at stimulation/F_PEAK_)/TC)] × 100 (Strojnik and Komi, 1998; Neyroud et al., 2014).

#### EMG data acquisition

Prior to MVC, the muscle belly was shaved and gently cleaned by abrasion with fine sandpaper and alcohol 70% at the anatomical point of the *vastus lateralis* muscle. The electromyograph Miotool 400 (Miotec - Porto Alegre–RS-Brazil) and bipolar electrodes of Ag/AgCl (3M-São José do Rio Preto–SP-Brazil) were aligned with the muscle fibers and positioned at a distance of 3 cm center-to-center. The electrode sites were ink demarked to allow placement reproducibility throughout the experiment. The EMG signal was acquired using a sample rate of 2000 Hz, an amplifier gain of 1000 and a band pass filter of 20–500 Hz. The mean Root Mean Square (RMS) of the 1-s force plateau, the peak-to-peak maximal amplitude evoked by the supramaximal electrical stimulus delivered to the relaxed muscle (M-Wave), and the ratio between RMS and M-Wave (RMS/MW) were calculated.

### Statistical analysis

Initially the normality of data distribution was confirmed by the Shapiro Wilk test. To verify the possible differences between halves of the simulated games and before and after the simulated games, the student *t*-test for dependent variables was used. To verify the possible differences between the sets of finishing kick performance an analysis of variance (ANOVA–one way) was used. The Pearson's correlation test was used to verify possible associations between the variables absolute variation in running performance (Δ = 2nd half–1st half: ΔDC, ΔDC_MIN_, ΔS_N_, and ΔS_T_), kick speed and accuracy (Δ = Set 3–Set 1, Set 3–Set 2, and Set 2–Set 1: Δ3–1; Δ3–2 and Δ2–1), physiological (Δ = 2nd half–1st half: Δ[La]_PEAK_, Δ[La]_MEAN_, ΔHR_PEAK_, and ΔHR_MEAN_) and neuromuscular outcomes (Δ = after–before: ΔF_PEAK_, ΔTC, ΔVA, ΔRMS, ΔM-Wave, and ΔRMS/MW). The significance level was assumed as 95% in all cases (*p* <0.05).

As an additional and qualitative analysis, the magnitude of differences between moments (i.e., game halves and sets of kicks) was calculated and expressed as standardized mean differences (Cohen's *d*), which were calculated using the pooled standard deviations of the two testing moments of interest (Cohen, 1988). Threshold values of effect size (ES) for Cohen's *d* statistics were > 0.2 (small), > 0.5 (moderate), and > 0.8 (large). Confidence intervals (95%) for the (true) differences were estimated (Hopkins et al., 2009). For between-moments comparisons, the chances that the true changes in performance were higher [i.e., higher than the smallest practically important effect, or the smallest worthwhile change, SWC (0.2 multiplied by the between-subject deviation, based on the Cohen's *d* principle)], similar, or lower, were calculated. The quantitative chances of positive or negative performance changes were assessed qualitatively as follows: ≤ 1% *almost certainly not*, > 1–5% *very unlikely*, > 5–25% *unlikely*, > 25–75% *possibly*, > 75–95% *likely*, > 95–99 *very likely*, > 99% *almost certain*. If the chance of having positive/beneficial or negative/harmful performances were both > 5%, the true difference was assessed as unclear (Hopkins et al., 2009).

## Results

**Figure 4:**
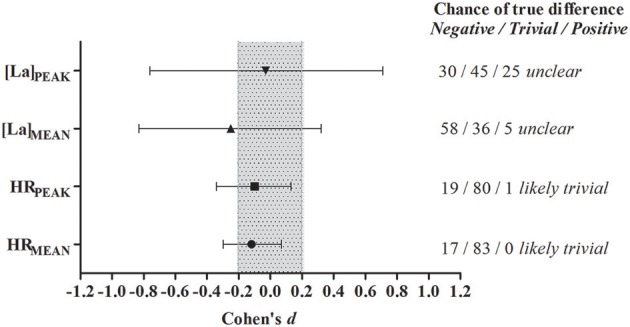
**Magnitude-based inferences of possible differences between halves of [La]_**PEAK**_, [La]_**MEAN**_, HR_**PEAK**_, and HR_**MEAN**_**. Shaded area represents the smallest worthwhile change. *N* = 10.

No differences were found between halves for [La]_PEAK_ (1st half: 5.6 ± 2.6 mmol.L^−1^; 2nd half: 5.5 ± 3.5 mmol.L^−1^, ES = −0.03, *P* = 0.93), [La]_MEAN_ (1st half: 4.8 ± 2.3 mmol.L^−1^; 2nd half: 4.2 ± 2.2 mmol.L^−1^, ES = −0.25, *P* = 0.34), HR_PEAK_ (1st half: 186.9 ± 9.2 bpm; 2nd half: 185.7 ± 10.0 bpm, ES = −0.10, *P* = 0.34), and HR_MEAN_ (1st half: 168.4 ± 12.4 bpm; 2nd half: 166.4 ± 12.5 bpm, ES = −0.12, *P* = 0.20). The magnitude-based analysis of blood lactate variables revealed *unclear* outcomes for the comparison between halves and confirmed that HR_PEAK_ and HR_MEAN_ were not altered during the game (i.e., *likely trivial* outcome) (Figure 4).

**Figure 5:**
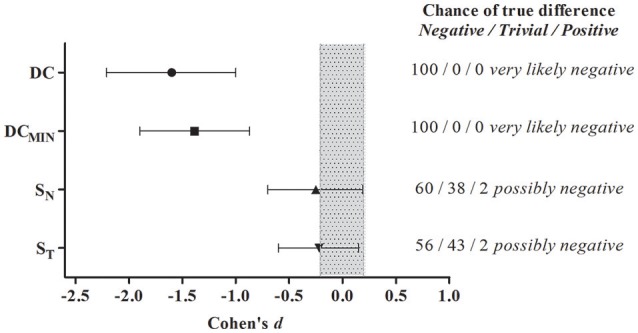
**Magnitude-based inferences of possible differences between halves of DC, DC_**MIN**_, S_**N**_, and S_**T**_**. Shaded area represents the smallest worthwhile change. *N* = 10.

The running performance analysis demonstrated significant differences between halves for DC (1st half: 1986.6 ± 74.4 m; 2nd half: 1856.0 ± 129.7 m, ES = −1.60, *P* = 0.00) and DC_MIN_ (1st half: 103.2 ± 4.4 m.min^−1^; 2nd half: 96.4 ± 7.5 m.min^−1^, ES = −1.39, *P* = 0.00), without a significant difference for S_N_ (1st half: 49.5 ± 14.5 a.u.; 2nd half: 45.5 ± 9.1 a.u., ES = −0.25, *P* = 0.23) or S_T_ (1st half: 70.6 ± 29.3 s; 2nd half: 63.4 ± 18.6 s, ES = −0.22, *P* = 0.21). Additionally, the magnitude-based analysis showed *very likely negative* outcomes for DC and DC_MIN_ and *possibly negative* outcomes for S_N_ and S_T_ (Figure 5).

**Figure 6:**
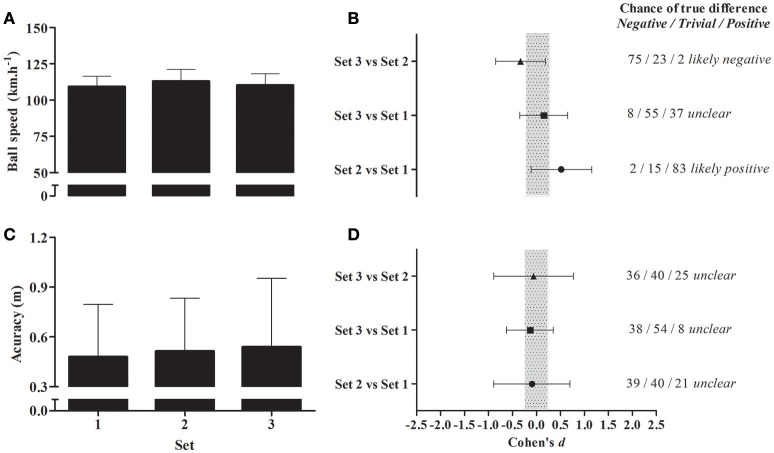
**(A)** Ball speed during sets of kicks. **(B)** Magnitude-based inference of possible differences between ball speed during sets of kicks. **(C)** Accuracy of sets of kicks. **(D)** Magnitude-based inference of possible differences between accuracy of sets of kicks. Shaded area represents the smallest worthwhile change. *N* = 10.

There were no significant differences between the sets of kicks for ball speed [Set 1: 109.3 ± 7.1 km.h^−1^; Set 2: 113.1 ± 8.1 km.h^−1^; Set 3: 110.2 ± 7.9 km.h^−1^, *F*_(2, 29)_ = 0.65, ES = 0.05, *P* = 0.53] and accuracy [Set 1: 0.48 ± 0.32 m; Set 2: 0.51 ± 0.32 m; Set 3: 0.54 ± 0.41 m, *F*_(2, 29)_ = 0.07, ES = 0.005, *P* = 0.93], however the magnitude-based analysis revealed higher ball speed for set 2 when compared to set 1 (i.e., *likely positive* outcome) and lower ball speed for set 3 compared to set 2 (i.e., *likely negative* outcome) with an *unclear* outcome for the comparison between sets 3 and 1. All outcomes for accuracy were *unclear* (Figure 6).

Significant decreases in F_PEAK_ (before: 840.2 ± 66.2 N; after: 751.6 ± 114.3 N, ES = −1.32, *P* = 0.04) and VA (before: 85.9 ± 7.5 %; after: 74.1 ± 12.3 %, ES = −1.33, *P* = 0.04) were observed, whereas the other neuromuscular variables did not differ statistically. Likewise, the magnitude-based analysis revealed *very likely negative* outcomes for F_PEAK_ and VA and *unclear* results for other variables (Table 1).

**Table 1:** **Mean ± SD of neuromuscular outcomes before and after a simulated game (***N*** = 6)**.

	**Before**	**After**	**Δ**	***P***	**ES**	**+ive**	**Trivial**	**−ive**	**Inference**
F_PEAK_ (N)	840.2±66.2	751.6±114.3	−88.6±80.1	0.04^*^	−1.32	2	0	98	*Most likely negative*
TC (N)	288.6±45.5	248.0±66.6	−40.6±54.4	0.12	−0.75	6	1	93	*Unclear*
VA (%)	85.9±7.5	74.1±12.3	−11.9±11.2	0.04^*^	−1.33	3	0	97	*Most likely negative*
RMS (mV)	0.53±0.14	0.49±0.16	−0.04±0.12	0.47	−0.22	21	4	75	*Unclear*
M-Wave (mV)	3.75±1.41	4.37±1.57	0.62±1.16	0.84	0.37	87	2	11	*Unclear*
RMS/MW(a.u.)	0.17±0.09	0.13±0.06	−0.04±0.05	0.70	−0.37	4	1	95	*Unclear*

*F_PEAK_, MVC peak force; TC, Twitch control; VA, Percentage of voluntary activation; RMS, Root mean square of the 1-s force plateau; M-Wave, Peak-to-peak maximal amplitude evoked by the supramaximal electrical stimulus; RMS/MW, Ratio between RMS and M-Wave*.

* *Significant difference (P <0.05)*.

The ΔF_PEAK_ (−88.6 ± 80.1 N) was significantly associated with ΔDC (−111.4 ± 70.3 m) (*r* = 0.83, *P* = 0.04) and ΔDC_MIN_ (−5.8 ± 3.6 m.min^−1^) (*r* = 0.83, *P* = 0.04). No significant correlations were found for physiological variables (−0.41 < *r* > 0.80, 0.06 < *P* > 0.81). or kick speed (−0.47 < *r* > 0.56, 0.24 < *P* > 0.99) and accuracy (−0.70 < *r* > 0.89, 0.15 < *P* > 0.89) (*N* = 6).

## Discussion

The aim of the present study was to investigate the influence of futsal match-related fatigue on running performance, neuromuscular variables, and finishing kick speed and accuracy. Running performance decreased during the simulated game and this was also the case for F_PEAK_ and VA. However, the finishing kick speed and accuracy were not significantly altered.

All variables of running performance were reduced during the simulated game, confirming the occurrence of the fatigue development, as stated in the literature (Barbero-Alvarez et al., 2008; Castagna et al., 2009; De Oliveira Bueno et al., 2014; Caetano et al., 2015; Dal Pupo et al., 2016). Furthermore, the DC_MIN_ was quite similar to that reported during official matches for Brazilian professional futsal players (1st half: 97.9 m.min^−1^; 2nd half: 90.3 m.min^−1^) (De Oliveira Bueno et al., 2014).

During the simulated game, the [La]_MEAN_ and HR_MEAN_ attained were ~74 and ~91% of the maximum values reached during the incremental test, characterizing the high energy demand of futsal. The repeated high-intensity activities with short recovery periods may have caused reduced running performance due to the accumulation of several metabolites and impairment in muscles contractile, especially at cross-bridge level (Debold, 2012). It is important to highlight that the accumulation of H^+^ ions (i.e., muscle acidosis), inorganic phosphate, and adenosine di-phosphate contribute to diminished Ca^2+^ sensitivity, lower restoration of adenosine tri-phosphate and consequently reduce the force level (Allen et al., 2008; Debold, 2012). However, metabolite accumulation possibly did not influence neuromuscular outcomes. The time needed to perform the final MVC of the last athlete evaluated, took up to 15 min, which is sufficient for reestablishment of muscle homeostasis, buffering H^+^ ions and consequently less interference of the afferent feedback mechanism that reduces the motor drive to the active muscles (Amann et al., 2011; Billaut et al., 2013).

The absence of investigations that have used the twitch interpolation technique in futsal makes comparison of our results difficult. The VA observed in the present study was slightly superior to Girard et al. (2013) (~80%) and slightly lower than Rampinini et al. (2011) (~90%), in addition to which F_PEAK_ was higher than that found by Girard et al. (2013) (~680 N). The significant decreases in F_PEAK_ and VA pointed to central fatigue, similar to the findings of Rampinini et al. (2011), but without significant changes in variables that indicate peripheral fatigue. Thus, one possible explanation could be the high environmental air temperature during the simulated games. Hyperthermia-induced by the environment contributes to VA decreases, affecting force production and characterizing central fatigue (Morrison et al., 2004; Nybo et al., 2014). According to Morrison et al. (2004), central fatigue arises when core temperature surpasses 38.5°C and this value could have been reached by the athletes during the simulated games. Duffield et al. (2012) verified mean core temperature of 38.8 ± 0.4°C after a simulated soccer game (3 × 20 min) performed with an environmental air temperature of 26.9°C and relative humidity of 65.0%. The environmental air temperature and relative humidity during the simulated games in the current study (30.9°C, 49.7%; 32.0°C, 44.4%; and 27.0°C, 46.7%, respectively) were higher than verified by Duffield et al. (2012). Taken together for an indoor court without an acclimatization system, it is possible to speculate that the environmental air temperature may have caused an important heat stress and consequently altered the core temperature of the athletes above 38.5°C, contributing to the decrement in F_PEAK_ and VA.

The statistically unchanged M-Wave amplitude and RMS/MW ratio suggest the maintenance of the transmission-propagation of the action potential along the sarcolemma (Racinais et al., 2007; Rampinini et al., 2011; Girard et al., 2013). The uncoupling between VA and RMS responses might be a result of the characteristic of the measures, since VA is the sum of the responses of all the knee extensor muscles to the superimposed motor nerve stimulation, while the EMG is a muscle-specific index (Girard et al., 2015b). Furthermore, it is possible that the load sharing strategies of *quadriceps femoris* could be altered under fatigue and contribute to maintenance of the RMS value of the *vastus lateralis* muscle after the simulated game (Bouillard et al., 2014).

Strong correlations were verified between ΔDC and ΔDC_MIN_ with ΔF_PEAK_, demonstrating that match-related fatigue is associated with the impairment of neuromuscular performance as illustrated by the decreases in VA (~14%) and F_PEAK_ (~11%) (Rampinini et al., 2011). These results are supported by Brocherie et al. (2014), whose findings verified a large prevalence of neuromuscular qualities in athletic performance for soccer players, especially during RSA activities. However, interestingly, the drop in neuromuscular performance did not affect kick speed or accuracy.

Our results for ball speed of finishing kick prior to the simulated games (i.e., set 1 − 109.1 ± 7.3 km.h^−1^) are higher than those found for elite (77.2 km.h^−1^) or semi-elite (71.9 km.h^−1^) futsal players from New Zealand (Naser and Ali, 2016) or amateur players from Brazil (87.4 km.h^−1^) (Barbieri et al., 2015). Despite the fact that the magnitude-based inference revealed *unclear* outcomes for kick accuracy, it was verified a slight increase of ball speed between sets 1 and 2 (i.e., *likely positive* outcome) may be linked to an increased muscle temperature (i.e., induced by both high environmental air temperature and exercise) that is apparently beneficial to muscle contractility during short bouts of high efforts, as well as finishing kicks (Girard et al., 2015a). On the other hand, the decrease of ball speed evidenced between sets 2 and 3 (i.e., *likely negative* outcome) might be indicative of fatigue influence in technical performance.

According to Barbieri et al. (2010) and De Witt and Hinrichs (2012), kick performance is a product of the linear and angular foot velocity and limb joint angles prior to and at the time of contact with the ball. Furthermore, elite players are able to perform fine adjustments during adverse situations (i.e., fatigue) while maintaining performance during the kicks (Barbieri et al., 2010). This could be the reason that significant differences in the kick speed and accuracy were not verified. In addition, Lyons et al. (2006) reported that the effects of fatigue induced by high-intensity exercise on performance skills dissipate quickly, ~2 min after cessation of exercise and this condition may have affected the measurements.

It is also important to note that several studies that verified impaired kick performance (i.e., speed and accuracy) used circuit tests (Ali et al., 2007; Currell et al., 2009; Russell et al., 2011; Radman et al., 2016) which, despite being acceptable, may not reproduce game demand. On the other hand, Rampinini et al. (2011) performed a simulated soccer game and verified central and peripheral fatigue factors immediately after the game (time-window for assessment >40 min) but without significant alterations in short-passing ability. Despite the different natures of short-pass and finishing kicks, this is another indication that neuromuscular fatigue may not significantly affect technical performance.

The main limitations of the present study, and consequently points for further investigations, are the absence of a direct measure of environmental air temperature, relative humidity and core and/or skin temperature. It is also important to attempt to reduce the time-window for neuromuscular assessment, since the neuromuscular fatigue condition is highly influenced by the recovery time, which could underestimate the central and peripheral fatigue variables (Taylor et al., 2009; Place et al., 2010). Finally, Aagaard et al. (2002) and Morel et al. (2015) verified that the rate of force development is a key factor of performance during movements with reduced contraction time, such as sprints and kicks (<250 ms), this way the F_PEAK_ could generate limited information due the different etiology between isometric contractions and kicks. Still, EMG and kinematic investigations during the finishing kicks should be addressed for a better understanding of futsal match-related fatigue and its influence on technical performance (i.e., kick speed and accuracy).

Thus, we conclude that futsal match-related fatigue influenced running performance, maximal isometric force of knee extension and voluntary activation. The association between neuromuscular and running performance variables demonstrates the need to consider force/power training in a futsal player training program to avoid an excessive drop in performance of these variables. Surprisingly, kick speed and accuracy were not significantly affected.

## Author contributions

Conceived and designed the experiments: FM, LV, RB, NN, Jd, PS, MP. Performed the experiments: FM, LV, RB. Analyzed data: FM, LV, RB, AZ, FB. Contributed reagents/materials/analysis tools: AZ, FB, Jd, PS, MP. Wrote the paper: FM, AZ, NN, MP.

### Conflict of interest statement

The authors declare that the research was conducted in the absence of any commercial or financial relationships that could be construed as a potential conflict of interest.

## Abbreviations

ANOVA: Analysis of variance
DC: Distance covered
DC_MIN_: Distance covered per minute
EMG: Surface electromyography
F_PEAK_: MVC peak force
HR_MEAN_: Mean heart rate
HR_PEAK_: Peak heart rate
*i*$\dot{\text{V}}{\text{O}}_{2\text{MAX}}$, Intensity attained to the $\dot{\text{V}}{\text{O}}_{2\text{MAX}};\;$ MVC: Maximum isometric voluntary contraction
M-Wave: Peak-to-peak maximal amplitude evoked by the supramaximal electrical stimulus
S_N_: Number of sprints
RER: Respiratory exchange ratio
RMS: Root mean square
RMS/MW: Ratio between RMS and M-Wave
S_T_: Time spent sprinting
TC: Twitch control
TS: Twitch superimposed
VA: Percentage of voluntary activation
$\dot{\text{V}}{\text{CO}}_{2}$: Carbon dioxide production
$\dot{\text{V}}{\text{O}}_{2}$: Oxygen uptake
$\dot{\text{V}}{\text{O}}_{2\text{MAX}}$: Maximum oxygen uptake
ΔDC.: Absolute variation between halves of distance covered
ΔDC_MIN_: Absolute variation between halves of distance covered per minute
ΔF_PEAK_: Absolute variation between moments of MVC peak force
[La]_MEAN_: Mean blood lactate concentration
[La]_PEAK_: Peak blood lactate concentration.
